# Supplementary material for: MetaRibo-Seq measures translation in microbiomes
Source: Nat Commun. 2020 Jun 29;11:3268. doi: 10.1038/s41467-020-17081-z (PMC7324362; doi:10.1038/s41467-020-17081-z)
Supplement: Supplementary file 10 — Supplementary Data 7 [file 41467_2020_17081_MOESM10_ESM.zip › File2/Confidence_VeryHigh_Taxonomy/306144_out.krona.html]

Javascript must be enabled to view this page.

members
magnitude
magnitudeUnassigned
count
unassigned
taxon
rank

306144\_out

5

5
2
superkingdom

976
phylum
5

5
200643
class

171549
order
5

5
family
815

5
816
genus


SRS016954\_contig\_number\_22962SRS020869\_contig\_number\_37928SRS148511\_contig\_number\_19663
species
329854
3

2292002
species
2

SRS012273\_contig\_number\_48084SRS012273\_contig\_number\_contig-100\_3002.78281
